# Lignin-Based Materials for Sustainable Rechargeable Batteries

**DOI:** 10.3390/polym14040673

**Published:** 2022-02-10

**Authors:** Han Young Jung, Jeong Seok Lee, Hyun Taek Han, Jaehan Jung, KwangSup Eom, Jung Tae Lee

**Affiliations:** 1 Department of Plant and Environmental New Resources, Kung Hee University, Yongin 17104, Korea; hy5880@khu.ac.kr (H.Y.J.); wdtjr2653@khu.ac.kr (J.S.L.); hthan@khu.ac.kr (H.T.H.); 2 Department of Materials Science and Engineering, Hongik University, Sejong 30016, Korea; 3 School of Materials Science and Engineering, Gwangju Institute of Science Technology (GIST), Gwangju 61005, Korea

**Keywords:** lignin, sustainable, energy storage, rechargeable battery, binder, separator, electrolyte, anode, cathode

## Abstract

This review discusses important scientific progress, problems, and prospects of lignin-based materials in the field of rechargeable batteries. Lignin, a component of the secondary cell wall, is considered a promising source of biomass. Compared to cellulose, which is the most extensively studied biomass material, lignin has a competitive price and a variety of functional groups leading to broad utilization such as adhesive, emulsifier, pesticides, polymer composite, carbon precursor, etc. The lignin-based materials can also be applied to various components in rechargeable batteries such as the binder, separator, electrolyte, anode, and cathode. This review describes how lignin-based materials are adopted in these five components with specific examples and explains why lignin is attractive in each case. The electrochemical behaviors including charge–discharge profiles, cyclability, and rate performance are discussed between lignin-based materials and materials without lignin. Finally, current limitations and future prospects are categorized to provide design guidelines for advanced lignin-based materials.

## 1. Introduction

**Figure 1:**
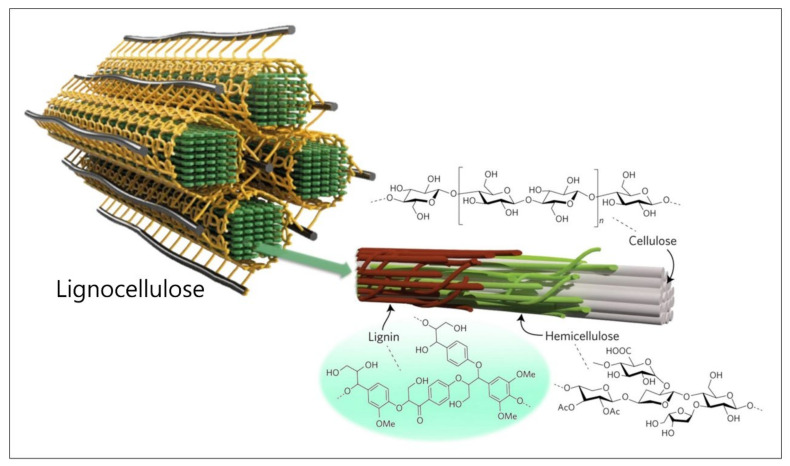
Schematic illustration of chemical and spatial structure of lignocellulose. Reproduced with permission from [11,12], Copyright (2020) Springer Nature, (2017) Springer Nature.

Lignocellulosic biomass, which is primarily constituted of lignin, cellulose, and hemicellulose, is one of the most naturally abundant resources on Earth [1]. Among the components in the lignocellulose, mechanically strong, biocompatible, and eco-friendly cellulose is being widely utilized in many applications such as paper, fiber, and the pharmaceutical industry [2,3]. Cellulose is isolated from the lignocellulosic biomass and used independently. Lignin is the byproduct of this process and is considered one of the main waste materials. Specifically, over 30 million tons of lignin is produced per year across the world, mostly obtained from kraft pulping [4,5]. As a significant amount (20–35 wt%) of lignin in the lignocellulosic biomass is renewable, harmless, and processible into functional materials due to its attractive functionalities, substantial research has been carried out to transform waste lignin into valuable materials in recent decades [6,7,8,9,10]. Lignin, the second-most prevalent natural polymer, chemically and spatially interacts with hemicellulose and cellulose fibers (Figure 1) [11,12]. It has complex amorphous three-dimensional (3D) macromolecules with different methoxylated phenylpropanoid units [5,13,14,15]. Furthermore, its functionalities such as hydroxyl, methoxy, carbonyl, carboxyl, and various aromatic rings enable various chemical production and modifications. 

The widespread use of lignin is a possibly efficient strategy to reduce environmental pollution. With the same logic, the use of electric vehicles (EVs) could positively impact the environment as it emits fewer greenhouse gases and air pollutants compared with conventional fuel-based vehicles. Energy storage is the key technology in EVs; hence, research on rechargeable batteries is being actively carried out [16]. Rechargeable batteries, as the name indicates, are reusable based on the reversible chemical reactions, and the most representative example is the Li-ion battery (LIB). Over the last decades, LIB technology has advanced tremendously, enhancing the energy density, cycle life, safety, and cost-efficiency [17,18,19,20,21,22,23]. Simultaneously, researchers actively pursue post-LIB technology such as solid-state, alkaline metal/ion (Na, K), and multivalent-ion (Mg, Ca, Al) batteries for further development [24,25,26,27,28,29,30]. Even though batteries are beneficial for the environment, many components in the battery are synthetic chemicals that are harmful to the environment during production and processing. If we partially replace these components with sustainable materials such as lignin, the energy storage or EV technology will be greener. As a result, lignin is processed or recycled for use in rechargeable batteries, and it shows great promise to improve the mechanical stability, ionic conductivity, thermal stability, redox-active material storage, and metal ion storage [31,32,33,34,35].

In this review, we will focus on the use of lignin in rechargeable batteries. The binder, separator, electrolyte, anode, and cathode are fundamental elements in rechargeable batteries, and lignin can be used for these components in different battery chemistries. In this paper, we will explain how and why lignin is employed in each component. Essential electrochemical properties, existing challenges, and visions of lignin-based materials for rechargeable batteries will be discussed.

## 2. Lignin-Based Binders, Separators, and Electrolytes

Lignin is a polyphenolic substance with an amorphous structure that is composed of hydroxycinnamyl alcohols (or monolignols) including p-coumaryl alcohol (H), coniferyl alcohol (G), and sinapyl alcohol (S). The basic lignin structures contain phenolic compounds and the C3 chain. The OH group in a benzene ring or C3 chain is the sole reaction site in lignin [36], and this is the main functional group for chemical modifications such as hydoxypropylation, esterification, alkylation, phenolation, etc. [14,37]. Adhesion capability, thermal stability, and enhanced ion transport can be achieved via the chemistry of lignin, and these properties are important factors for designing battery binders, separators, and electrolytes. Furthermore, the lignin is predominantly connected by ether bonds (e.g., β-O-4, α-O-4, and 4-O-5) and C-C bonds (e.g., β-5 and 5-5) between phenylpropanoids, resulting in a variety of connections, including aryl linked monolignol units. Among them, the *β*-O-4 ether bond is the main connection in lignin and is responsible for 40–65 percent of entire linkages based on the source of biomass [38]. The highly condensed linkage of lignin offers high mechanical strength and rigidity, which are also crucial features in binders and separators. These traits of lignin provoke tremendous research and development of lignin-based binders, separators, and electrolytes for rechargeable batteries [39,40,41].

### 2.1. Lignin-Based Binder

**Figure 2:**
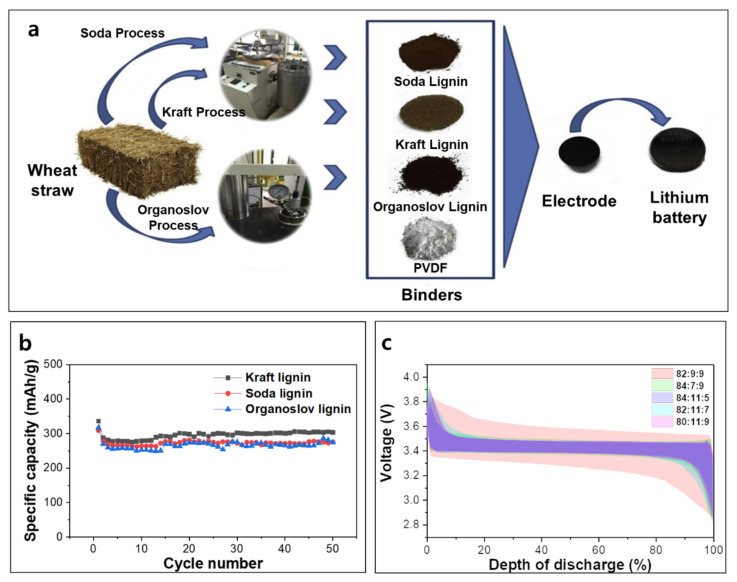
(**a**) Schematic illustration of battery preparation with lignin-based binders made by different manufacturing processes; (**b**) cycle performance of cells with lignin-based binders and PVDF at C/4; (**c**) initial charge–discharge profiles of Si cells with different compositions at C/10. The compositions between Si, conductive additive, and lignin-based binder are 82:9:9, 84:7:9, 84:11:6, 82:11:7, 80:11:9 by weight. Reproduced with permission from [40,43], Copyright (2016) MDPI, (2017) Elsevier.

The main function of the binder in a rechargeable battery is binding the active material, conductive additive, and current collector to build an electrode without sacrificing electron and ion transport. Conventionally, the binder is a polymeric material and is dissolved into the solvent to prepare a slurry. Polyvinylidene fluoride (PVDF), carboxymethylcellulose (CMC), and styrene-butadiene rubber (SBR) are common binder materials [42]. Similarly, lignin can be employed as a binder material by dissolving it into a polar solvent such as N-methyl-2-pyrrolidone (NMP). Lignin would be of great importance in the binder as it can provide binding capability with good mechanical strength and electrochemical stability. To investigate electrochemical behaviors of cells fabricated using lignin-based binders, three different types of lignin prepared by soda, kraft, and organosolv processes were mixed with 85 wt% mesocarbon microbeads (MCMB), 10% binder, 5% Super P, and NMP to fabricate electrodes [43]. All lignin-based cells with a LiPF_6_-based electrolyte showed stable cycling performance at C/4 over 50 cycles with similar discharge capacities (Figure 2). Interestingly, kraft lignin-based cells demonstrated an improved rate performance than those with the other two kinds of lignin and even more than those with PVDF binders. In contrast, the other two lignin (i.e., by soda and organosolv process) showed a similar or inferior rate performance to PVDF binders. The origin of this phenomenon was explained by the fact that the fraction of hemicellulose in kraft lignin is lower than the other two kinds of lignin. The influence of the lignin fraction in the electrode is also systematically studied [40]. The full cell comprising lithium iron phosphate (LFP) cathode with lignin binder, graphite anode, and electrolyte consisted of 1 M LiPF_6_ with a 1:1 mixture of EC and DEC was prepared. The ratios between LFP, Super P, and lignin were 84:7:9, 82:9:9, 80:11:9, 82:11:7, and 84:11:5 and the electrode with 80:11:9 ratio showed the best performance among investigated samples. It demonstrated a discharge capacity of ~148 mAh g^−1^, similar to the conventional electrode employing PVDF binder, and showed good capacity retention up to 50 cycles. LFP electrodes with different compositions show almost identical charge–discharge profiles, except for the electrode with an 82:9:9 ratio, which shows a significantly larger polarization (Figure 2c). 

**Figure 3:**
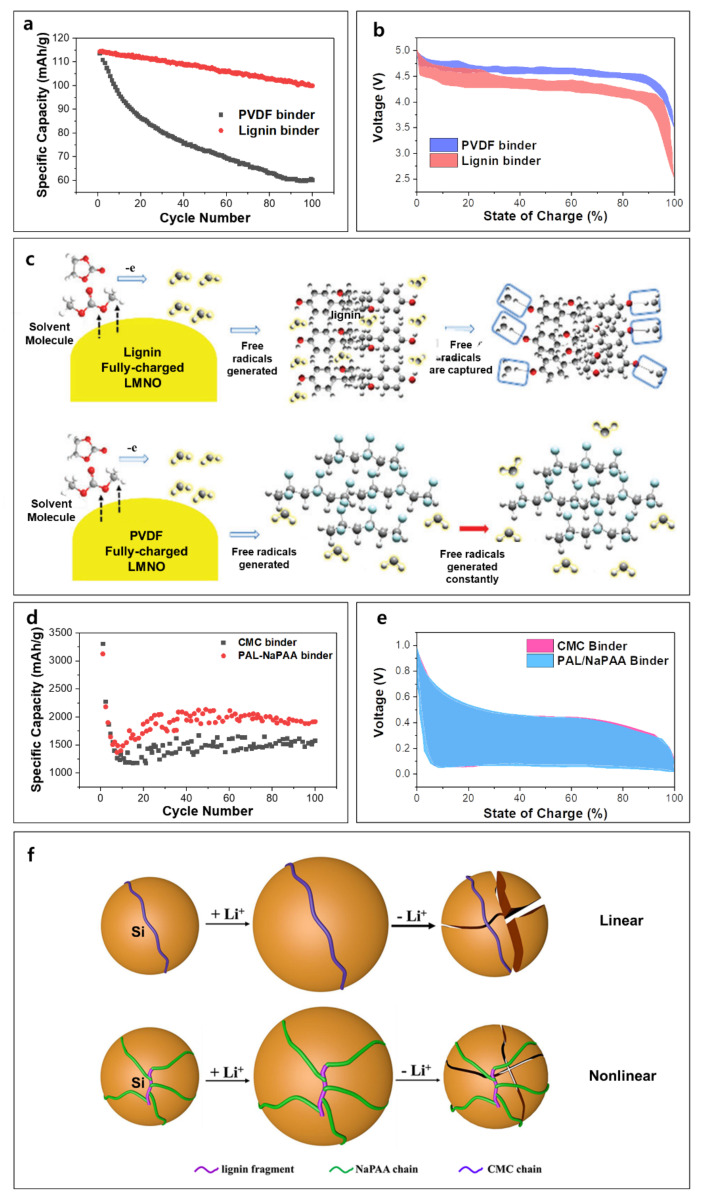
Electrochemical characterization and schematic illustration of cells with/without lignin-based binder: (**a**) cycle performance and (**b**) initial charge/discharge profiles of LiNi0.5Mn1.5O4/graphite cells with lignin-based binder and PVDF at 150 mA g^−1^, (**c**) schematic illustration of free radical scavenging ability of lignin, (**d**) cycle performance and (**e**) initial charge/discharge curves of Si/Li cells with lignin-based (PAL/NaPAA) binder and CMC at 840 mA g^−1^, and (**f**) schematic illustration of nonlinear binding of lignin in LIBs. Reproduced with permission from [33,47], Copyright (2019) Royal Society of Chemistry, (2018) American Chemical Society.

The lignin-based binder provides extra functionalities in different battery chemistries. To analyze the impact of lignin on the electrochemical behaviors in detail, the capacity retention and charge–discharge profiles of LiNi0.5Mn1.5O4/graphite cell with lignin-based and PVDF binders are compared (Figure 3a,b) [33]. The cell with lignin shows noticeably improved cycling performance than the cells with PVDF at 1 C over 100 cycles. However, the discharge voltage plateau was ~0.4 V lower (at 50% of the state of charge) when a lignin binder was used (Figure 3b). This indicates that the lignin binder deteriorates ion and/or electron transport in the electrode, resulting in a decrease in energy density. The reason for the improvement in cyclability is that lignin impedes the activity of free radicals [33]. Free radicals derive massive decomposition of electrolytes, leading to the formation of unstable cathode electrolyte interphase. Carbonate-based solvents and lithium salts react continuously, generating free radicals at cathode–electrolyte interfaces [44]. However, as shown in Figure 3c, abundant –OH groups in lignin suppress these undesired reactions by capturing free radicals [33,45].

Lignin can also show effective binding ability for conversion-type materials which experience high volume expansion during electrochemical reactions. The Si is a very promising anode material because of its low price plus outstanding theoretical capacity of 4200 mAh g^−1^. However, the volume change of Si can be up to 300% during lithium insertion and extraction, and this causes fast capacity fade due to crack formation [46] Lignin-grafted sodium polyacrylate (PAL−NaPAA) binder for a micro-silicon anode was synthesized by alkaline hydrolysis of PAL-polyacrylonitrile (PAN), which is derived from alkali lignin [47]. The electrode was prepared by mixing Si, Super P, and PAL-NaPAA with a ratio of 6:1:1 by weight. This electrode was paired with Li metal with LiPF_6_-based electrolyte. Si with a lignin-based binder retained 40% higher capacity after 100 cycles compared with the cell without a lignin-derived binder (Figure 3d) [48]. Based on the galvanostatic charge–discharge profile comparison, both electrodes with/without a lignin-derived binder showed almost identical polarization (Figure 3e). The lignin-derived binder provides nonlinear binds at the anode, suppressing crack generation, leading to enhanced electrochemical properties, as illustrated in Figure 3f.

### 2.2. Lignin-Based Separator

**Figure 4:**
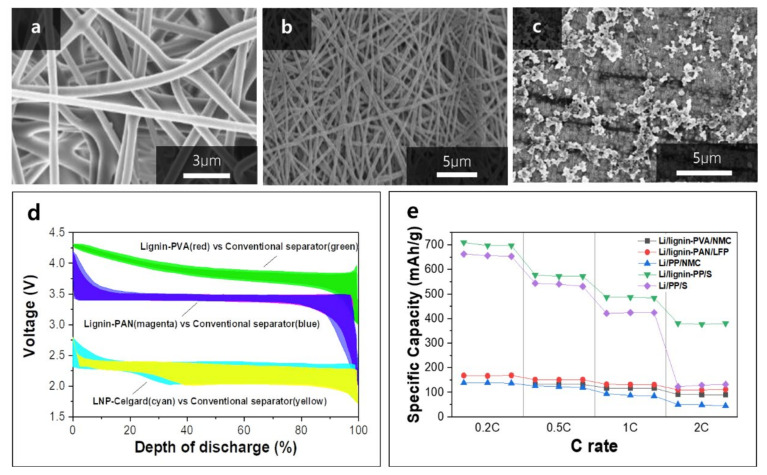
The SEM micrographs of (**a**) lignin–PVA, (**b**) lignin–PAN, (**c**) LNP–Celgard, and (**d**) initial charge/discharge profiles of lignin–PVA, lignin–PAN, and LNP–Celgard at 0.1 C, 0.2 C, and 1 C, respectively, and (**e**) rate performance of different cells with/without lignin-based separators. Reproduced with permission from [32,39,56], Copyright (2015) Royal Society of Chemistry, (2017) Elsevier, (2019) MDPI.

The separator is another key component in the rechargeable batteries, while it does not participate in electrochemical reactions [49]. It separates the anode and cathode to prevent direct contact, enabling electron flow in the external circuit. In alkaline metal and metal-ion batteries, dendrites formed at the electrode surface can cause internal short-circuiting, resulting in serious safety issues such as thermal runaway, gas generation, and explosion [3,50,51,52,53]. Polyolefin-based membranes (i.e., PP, PE) are broadly used in the battery industry owing to their excellent mechanical strength and electrochemical stability [54]. However, poor thermal stability and ion conductivity have to be further improved for the next-generation rechargeable batteries. Lignin is a promising separator material as its membrane has great thermal stability, ionic conductivity, and mechanical strength [55]. The ionic transport arises from the ions in the liquid electrolyte. The separator holds this liquid electrolyte in its pores; hence, the pore properties of the separator such as pore size, porosity, and pore size distribution determine the overall ionic conductivity in the cell. To obtain desirable pore properties and mechanical strength simultaneously with lignin, constructing a separator in nanofiber is an efficient strategy. Lignin nanofibers synthesized by electrospinning/melt extrusion process are usually used to prepare separators with polymer additives. As an example, lignin–polyvinyl alcohol (lignin–PVA) film was produced by the electrospinning method by mixing lignin and PVA with a ratio of 1:1 by weight [39]. As shown in Figure 4a, the lignin–PVA membrane shows three-dimensional entanglements and large pores accounting for excellent mechanical property and high electrolyte uptake, respectively. Similarly, lignin–polyacrylonitrile (lignin–PAN) separators were prepared via electrospinning of lignin and PAN mixture with 0:10, 1:9, 3:7, and 5:5 ratios by weight [32]. The Lignin–PAN membrane also shows high porosity, as shown in Figure 4b, and the porosity is increased with an increasing fraction of lignin. The lignin nanoparticle (LNP) coated on a conventional separator was applied in a Li-S battery to reduce polysulfides shuttle and facilitate Li-ion transport [56]. The Li-S battery is an encouraging next-generation energy storage system because of its high energy density and cost efficiency [57]. However, the polysulfide dissolution and its movement between cathode and anode (shuttle effect) are regarded as the main challenge. This problem can be suppressed by introducing linin in the separator as the electron-donating groups in lignin repulse negative polysulfide ions. LNP-coated Celgard (LC) was prepared by vacuum filtration with ethanol-dispersed LNP. Nano-sized lignin with an average size of ~100 nm was attached to the exterior pores of Celgard (Figure 4c). Electrochemically, Li/Li(Ni_0_._33_Mn_0_._33_Co_0_._33_)O_2_ cells with lignin–PVA and PP-based separators show an almost similar discharge capacity at 0.2 C; however, the polarization in the cell with lignin–PVA separator is noticeably reduced, indicating higher energy efficiency (Figure 4d, red and green). Moreover, the lignin–PVA separator demonstrates improved rate performance from 0.5 C to 2 C (Figure 4e). The Li/LiFePO_4_ cell with lignin–PAN (3:7 wt%) separator among all lignin–PAN separators demonstrated the best discharge capacity of 156.9 mAh g^−1^ at 0.2 C, while cells with 1:9 wt% and 5:5 wt% showed 154.6 mAh g^−1^ and 112.7 mAh g^−1^, respectively. The discharge capacity of a cell with a conventional separator was around 137.5 mAh g^−1^. Between the cells with and without the lignin separator, no significant polarization change was observed at 0.2 C (Figure 4d, magenta and blue). However, the lignin–PAN separator demonstrated improved rate performance from 0.2 C to 2 C compared with the conventional separator (Figure 4e). The Li/S cells with a lignin-coated separator show quite different charge–discharge profiles (Figure 4d, cyan and yellow). Firstly, the length of the upper discharge plateau and the following sloping region which originates from the soluble polysulfides is shorter when a lignin-coated separator is used in the cell. Additionally, the voltages of the upper discharge plateau are exactly matched, but the cell with a lignin-coated separator demonstrates slightly higher discharge voltage, indicating higher energy density. Interestingly, the overall voltage of the charge plateau is higher with the lignin-coated separator, indicating that the addition of lignin requires more energy to be charged.

The fundamental mechanistic study may provide important roles of lignin-based components in the Li-S chemistry. A lignin-coated separator in Li/S cell demonstrates higher capacities than its counterpart at all tested rates, and especially at 2 C, the capacity difference became almost doubled (Figure 4e). 

### 2.3. Lignin-Based Electrolyte

**Figure 5:**
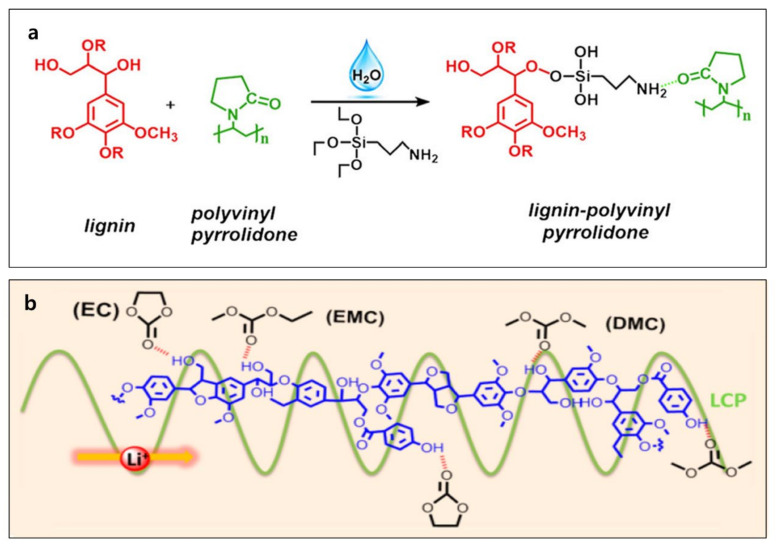
Preparation of (**a**) lignin–PVP and (**b**) lignin–LCP gel electrolytes. Reproduced with permission from [68,69], Copyright (2018) Springer, (2018) American Chemical Society.

The electrolyte in a rechargeable battery is a medium in which ions move between cathode and anode without flowing electrons [58]. Like the binder and separator, it is not a redox-active material, but it is a key component to obtain a functional battery. The ions can move in both liquid and solid. As a result, electrolytes in the battery can be liquid, solid, and a mixture (gel). Thus far, lignin is mainly applied in the solid polymer and gel electrolytes to suppress Li dendrites, active material dissolution, and dissolved material shuttle [59,60]. Lignin-based solid and gel electrolytes which are biodegradable and sustainable can positively influence the environment by replacing traditional polymers such as PVDF, polymethyl methacrylate (PMMA), and polyethylene oxide (PEO) [59,61]. The lignin-derived graft polymers prepared by chemical modification and atom transfer radical polymerization offer ion conduction paths and suppression of the lithium dendrite formation, which is the main problem in the Li metal battery [55,62,63]. The use of Li metal in the rechargeable battery system was abandoned due to the safety issue; however, currently, researchers are aiming to revive the employment of Li metal because Li metal has an ultra-high energy density based on the low density, high theoretical capacity, and low reduction potential [64,65,66]. The positive impact of lignin in the metal battery is confirmed [53,55,67], but detailed mechanistic studies are needed in the future. The lignin–polyvinylpyrrolidone (PVP) gel electrolyte was synthesized by adding silanol and PVP to the neutralized alkaline lignin slurry (Figure 5a) [68]. Li/LFP cell with lignin–PVP demonstrated ionic conductivity of 2.52 × 10^−3^ S cm^−1^ at ambient temperatures, which is much higher than the combination of the commercial separator and liquid electrolyte (0.21 × 10^−3^ S cm^−1^). The cell with lignin demonstrated a similar capacity retention (80% over 100 cycles) and improved rate performance (15% higher capacity at 2 C) compared with the cell with a PP separator. The robust lignin–linear poly(N-vinyl imidazole)-co-poly(poly(ethylene glycol) methyl ether methacrylate) copolymer (LCP) gel electrolyte was fabricated by a casting method (Figure 5b) [69]. LCP demonstrates reasonable film-forming capability, promising electrochemical performance, and good solubility in water for good binding with the lignin via a physical crosslinking network. Electrochemically, the cell with lignin-based gel electrolytes shows improved specific capacities at different current densities, validating the promise of lignin-based gel electrolytes.

## 3. Lignin-Based Anodes

The anode where redox reactions occur is the negative terminal during discharge in a rechargeable battery. Carbon materials are well-known redox-active anode materials with good electronic conductivity in rechargeable batteries [70,71]. Redox reactions are chemical reactions that involve electron transfer; hence, good electronic conductivity is the key parameter to design advanced anode materials. Graphite is the approved anode material in LIBs. However, graphite has an unsatisfactory specific capacity, which is incompatible with the future applications demanding high energy and power densities, such as unmanned aerial vehicles and electric vehicles. Significant efforts have been made on the amorphous carbon materials with/without porous structures to improve the energy storage abilities. Amorphous carbons generally demonstrate high specific capacities and rate performance based on their controllable porous and disordered structures that make it easy to diffuse metal ions [72,73,74]. Porous carbon materials also exhibit attractive energy storage capability since the pores in carbon can result in enhanced reversibility of redox reactions and area of reaction site and reduced ion diffusion length. Cost-effective and environmentally friendly lignin may be a crucial source for the carbon materials for the anode. The pyrolysis process can convert the lignin into carbon structures by cleaving linkages between the lignin units between 473 and 673 KDa [75]. Pyrolysis of lignin is the thermal degradation across a wide temperature range, resulting in 30–50% of hard carbon (or char) and a large number of low molecular mass volatiles. The formation of char strongly depends on the thermolysis temperature and higher product yields, are obtained at higher temperatures [76,77]. The combination of carbon-centered radicals yields the creation of strong C-C bonds including the full rearrangement of the carbon skeleton. Lignin is an ideal carbon source for generating high-performance carbon compounds such as graphene due to its unusual polymeric structure [5,31,78]. A carbon-rich network of phenolic monolignols results in a high yield of carbon after the pyrolysis [78,79]. The network of aromatic rings in lignin forms graphite-like structures. The pore structure can be introduced by physical or chemical treatments [80]. Forming a graphite-like structure, porous carbon, and foreign element-doped carbon (N, S, P) derived from lignin promotes the ion kinetics, electron transport, and structural stabilization of rechargeable batteries [81,82,83,84]. 

**Figure 6:**
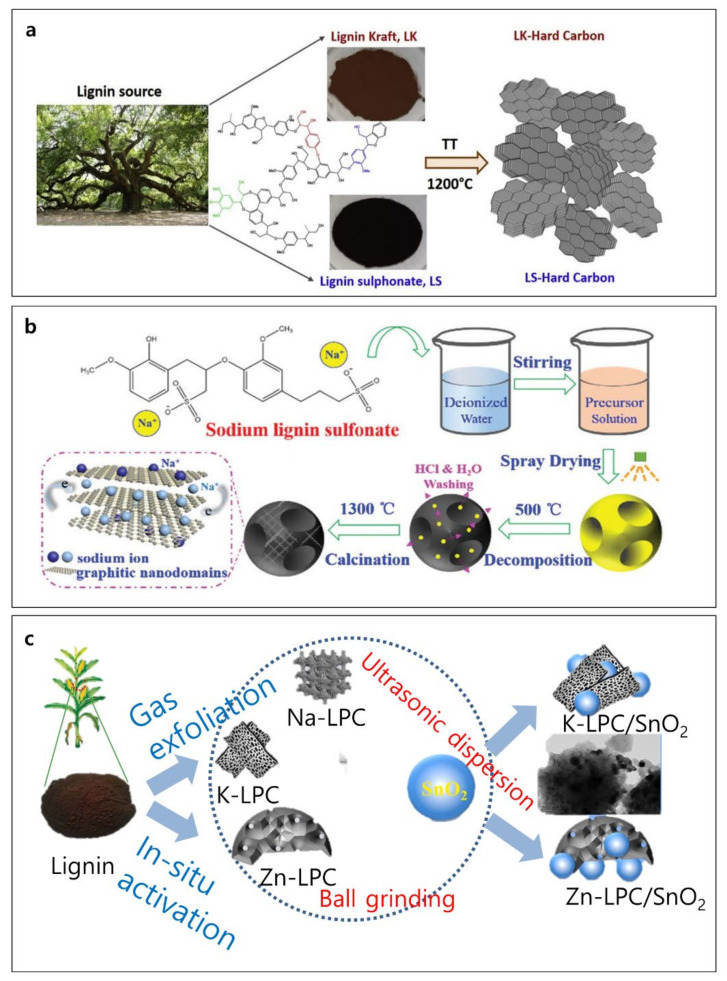
Schematic illustrations of lignin-based hard carbon: schematic diagrams of (**a**) hard carbon derived from lignin kraft (LK) and lignin sulphonate (LS), (**b**) hard carbon microspheres (HCMs) from a single sodium lignin sulfonate source sodium-ion storage, and (**c**) LPC/SnO_2_ composite preparation process for Li-ion batteries. Reproduced with permission from [84,86,88], Copyright (2019) Elsevier, (2020) Royal Society of Chemistry, (2021) Elsevier.

Lignin-derived hard carbons have been extensively utilized as an anode material in the field of Na-ion battery (NIB). Since sodium ion with a size of 2.04 Å is difficult to intercalate into graphite with an interlayer distance of 1.86 Å, expanded interlayer distance is required for Na ions to be electrochemically intercalated. For Na ion insertion, an interlayer distance of 0.37 nm is thought to be necessary [85]. Lignin kraft and lignin sulphonate were studied as promising sources to produce hard carbon for NIB (Figure 6a) [86]. The interlayer distances of kraft lignin and lignin sulphonate-derived carbons are ~3.842 Å and ~3.827 Å, respectively, and these values are enough for the intercalation of Na ions. Hard carbons with varied surface chemistry, shape, and porosity were formed after thermal annealing (1200 °C). The surface areas of lignin sulphonate-derived hard carbon (sample termed LS-HC) and lignin kraft-derived hard carbon (sample termed LK-HC) differs significantly (180 m^2^ g^−1^ for LS-HC vs. 1.8 m^2^ g^−1^ LK-HC). Electrochemically, the LK-HC shows almost constant capacities around 181 mAh g^−1^ for 50 cycles, whereas the LS-HC demonstrates an initial discharge capacity of 205 mAh g^−1^ with quick capacity fade after 30 cycles. The origin of this phenomenon is that the huge surface area of LS-HC facilitated the capacity loss during electrochemical cycling. The thorough washing of LS significantly reduced contaminants, allowing for an effective reduction in specific surface area (5.6 m^2^ g^−1^). The cleaned LS-HC demonstrated the highest capacity of 284 mAh g^−1^ and 78.1% retention for 50 cycles [86]. The large fraction of benzyl alcohol groups in sodium lignin sulfonate renders it a suitable source for hard carbon. The aromatic components of sodium lignin sulfonate promote a great yield of graphitic segments, which is generally associated with superior conductivity [87]. A hard carbon microsphere (HCM) is created using simple spray drying and carbonization (Figure 6b) [88]. HCM achieves an interlayer distance of 0.396 nm, an initial capacity of 339 mAh g^−1^, and capacity retention of 93% after 100 cycles in NIB. The HCM is dense, resulting in fewer surface defects, a smaller surface area, and a lower SEI production rate. Furthermore, the increased interlayer spacing and organized graphitic nano-domain facilitate reversibility [88]. Lignin-derived hard carbons were also applied in a K-ion battery, which is another promising next-generation battery system based on the high energy density and low cost [89]. In this study, the molecular weight of lignin and carbonization temperature influenced the potassium ion storage, and among all samples, lignin with a molecular weight of 9600 g mol^−1^ carbonized at 700 °C demonstrated the highest specific capacity of ~300 mAh g^−1^ at 50 mAg^−1^. Although the lignin-derived carbon material itself is a promising anode material, different anode materials can combine with lignin-derived carbons to achieve high-capacity anodes with enhanced cyclability. As an example, tin dioxide (SnO_2_) has a large theoretical capacity of 1494 mAh g^−1^ and a stable crystal structure. The lithiation/de-lithiation of tin results in a 300% volume change and the continual generation of the SEI layer, leading to a rapid capacity fade. These problems could be mitigated by lignin-based porous carbons (LPCs), having a high specific surface area, graphitization, and hierarchical porosity (Figure 6c) [84]. The hierarchical porous LPCs enhance electron/ion access to SnO_2_ nanoparticles by overcoming severe repeated volume changes, leading to a dramatic improvement in specific capacity (64 vs. 620 mAh g^−1^).

Lignin can also produce binder-free anodes with short diffusion paths and fast reaction kinetics [90]. Carbon fibers [91,92] have broad applications in the automotive, aerospace, and electronics industries. Pitch, obtained from coal or petroleum, and polyacrylonitrile have been popular precursor materials to manufacture commercial carbon fiber since the 1960s. PAN is a petroleum-derived linear polymer with polar nitrile groups which are responsible for the high mechanical characteristics of carbon fibers based on strong intermolecular interactions. Carbon fiber made from PAN is costly; hence, its use is limited (i.e., high-performance structural components). Lignin is a functionally, environmentally, and economically appealing alternative to PAN to synthesize carbon fibers. Although PAN-based carbon fibers are often made by wet- or dry-spinning, oxidative stabilization, and carbonization, lignin-based carbon fibers are typically prepared via electrospinning [81,85] or melt-spinning [79]. 

**Figure 7:**
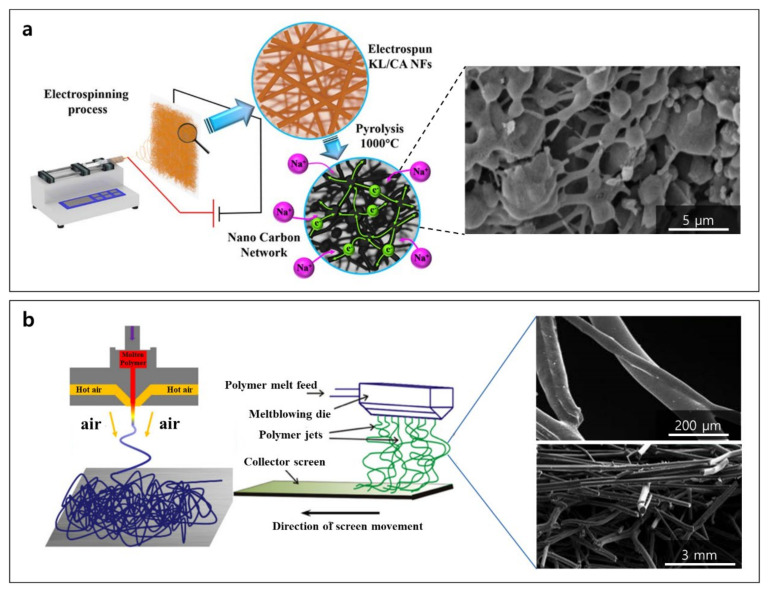
Preparation of binder-free carbon fiber electrodes: (**a**) schematic diagrams of fabrication procedure of carbon fiber with electrospinning process and an SEM micrograph of E-KL/CA-C (electrospun kraft lignin/cellulose acetate-derived nanocarbon network), and (**b**) schematic illustration of melt blowing process and SEM micrographs of fused lignin carbon fiber mat of phthalic anhydride-modified kraft softwood lignin. Reproduced with permission from [79,85,94], Copyright (2018) American Chemical Society, (2014) American Chemical Society, (2019) American Chemical Society.

Electrospun lignin-based carbon nanofibers might provide lower charge-transfer resistance and a high number of reaction sites. Due to its high polydispersity indexes and low molecular weight, the chain structures and molecular entanglements of KL are reduced [93]. Due to its unique chemical structure as well as abundant availability and inexpensive cost, cellulose acetate (CA) was chosen as the complementary carbon source to KL. After carbonization (1000 °C) in an inert environment, non-woven nanocarbon fiber electrodes are made from mixed precursors of kraft lignin (E-KL) and cellulose-acetate-derived nanocarbon (CA-C) (Figure 7a) [85]. The electrospun oxygen-rich lignin-based carbon membrane prepared by UV/ozone treatment could successfully host Li metal by uniform Li metal deposition [53]. The effects of lignin source alteration on melt-processing and the induced carbon microstructures were investigated (Figure 7b) [79,94]. Softwood lignin has more cross-linking, which caused high viscosity and needs more pressure for extrusion. Unlike softwood lignin, hardwood lignin has less cross-linking, which is easy to extrude, for electrospinning. Softwood lignin was modified to provide similar functionalities and rheological characteristics of hardwood lignin, allowing for melt-processing of softwood lignin in the air. Unmodified/acid anhydride-modified Al cell hardwood lignin (AHL) and kraft softwood lignin (KSL) were melt-extruded and spun into lignin fibers. Modified softwood becomes a similar structure to unmodified hardwood. Unmodified AHL and acid anhydride-modified KSL have a hollow center and porous surface. These structures gives rise to the increase in interface between the electrolyte and anode [79].

**Figure 8:**
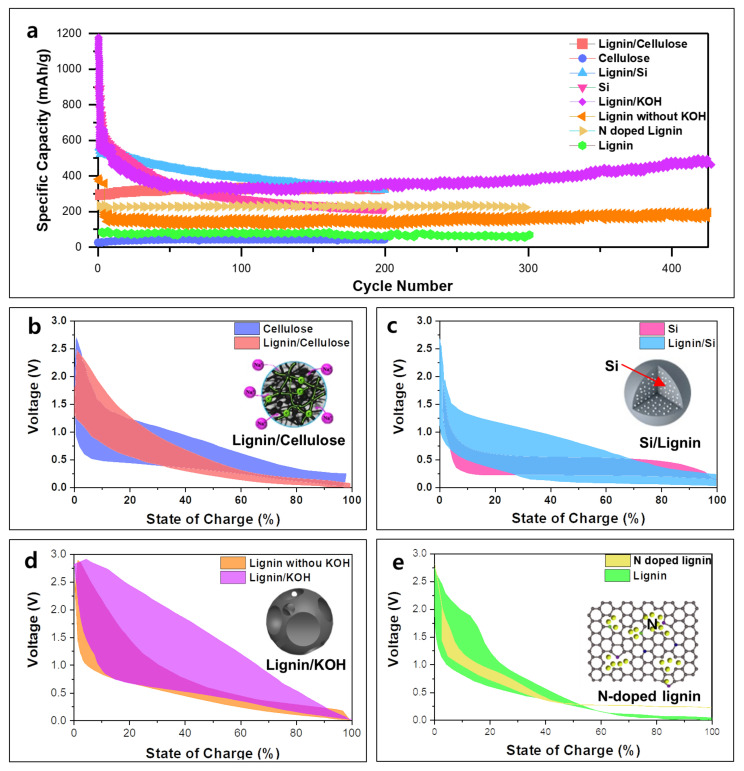
Electrochemical performance of lignin-based anode and non-lignin-based anode: (**a**) cycle performance of the lignin-derived carbon anodes and lignin-free anodes, and charge–discharge profiles of (**b**) carbonized cellulose and lignin/cellulose (E-KL/CA-C, E-CA-C) at 50 mA g^−1^, (**c**) Si and lignin/Si(Si with lignin-derived carbon composite) at 0.1 A g^−1^, (**d**) carbonized lignin with/without KOH treatment at 200 mA g^−1^, and (**e**) carbonized lignin with/without nitrogen doping 25 mA g^−1^. Reproduced with permission from [83,85,98,99], Copyright (2018) American Chemical Society, (2020) American Chemical Society, (2015) Elsevier, (2021) Elsevier.

The electrochemical behaviors of various lignin-derived anode materials as well as lignin-free anode materials are compared (Figure 8). The electrode prepared from the mixed KL and CA (sample termed “Lignin/Cellulose”) had a carbon network with an interplanar spacing of 0.384 nm, oxygen content of 13.26%, and a specific surface area of 540.95 m^2^ g^−1^. The nanocarbon network structure gives a specific capacity of 290 mAh g^−1^ with a coulombic efficiency of 52% in the first cycle and the increased capacity of 340 mAh g^−1^ with a coulombic efficiency of 99% after 200 cycles at a current density of 50 mA g^−1^. The electrode derived from CA (sample termed “Cellulose”) demonstrated approximately 30 mAh g^−1^ lower initial capacity and coulombic efficiency of 46% after 200 cycles. The carbon network produced from KL/CA nanofibers retained sections of the fibrous structure, enabling facile electron and ion transport [85]. The pore structure of the anode can facilitate the transportation of Li ions and enlarge the surface area for fast reaction kinetics. Lignin-derived hierarchical porous carbon (LHPC) can be prepared by mixing alkali lignin and KOH followed by heat treatment (sample termed “Lignin/KOH”). Through KOH treatment and carbonization, the lignin/KOH sample can have macro-, meso-, or micropores, and a retained capacity of 470 mAh g^−1^ until 400 cycles at a current density of 200 mA g^−1^. In contrast, the lignin-derived carbon without chemical activation (sample termed “Lignin without KOH”) achieved 180 mAh g^−1^ after 400 cycles.

Lignin-derived carbon can host different redox-active materials, and due to its excellent theoretical capacity and competitive price, silicon (Si) is one of the favorable anode solutions for next-generation LIBs. Unfortunately, the electrical conductivity of Si is not satisfactory and Si anodes have high volume change during the lithiation–delithiation process. A coprecipitation process of Si/lignin composite and following annealing forms Si-nanoparticle-loaded carbon particles (sample termed “Si/Lignin”), which would overcome the intrinsic problems of Si materials, such as significant volume change during electrochemical reactions and low conductivity. The initial charge capacity of the Si/Lignin sample was 1016.8 mAh g^−1^ with high-capacity retentions of 74.5% and 57.5% after 100 and 200 cycles, respectively, at 0.2 A g^−1^ [83]. In the lignin-based carbon structure, abundant -OH groups of lignin lead to a negative charge for lignin composite and cationic surfactant CTAB is added, which makes Si particles become a positive charge. As Si particles and the electrolyte do not directly touch by encapsulated carbon matrix, the Si particles are effectively contained with carbon composite by electrostatic attraction. As a result, the lignin-derived carbon matrix in the Si/lignin sample enables overall structural stabilization and pulverization suppression during cycling.

The surface functionalities (such as P and N) can improve the electrochemical performance of carbons [95,96,97]. Nitrogen-doped lignin-based carbon (sample termed “n-doped Lignin”) can be obtained by carbonization with a 3-aminophenol (nitrogen source) [98]. N-doped lignin has a small graphitic region, significant micro-, meso-, and macro pores, and enlarged interlayer space. Lignin-based carbon without n-doping (sample termed “undoped Lignin”) has a lower specific area and pore volume than n-doped lignin-based carbon (area: 531.1 m^2^ g^−1^ vs. 727.4 m^2^ g^−1^, pore volume: 0.158 m^3^ g^−1^ vs. 0.215 m^3^ g^−1^). N-doped carbons display a high reversible specific capacity of 374 mAh g^−1^ at 25 mA g^−1^ and excellent capacitance retention of 90% after 300 cycles at 100 mA g^−1^ with stable Na ion intercalation/adsorption.

## 4. Lignin-Based Cathodes

**Figure 9:**
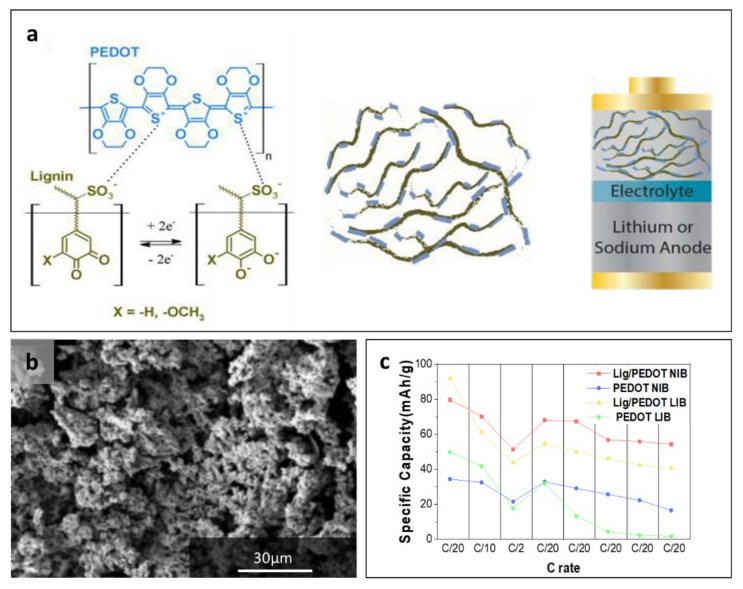
(**a**) Structure of lignin/PEDOT for NIB and LIB, (**b**) SEM micrograph of lignin/PEDOT (20/80 by weight), and (**c**) capacities of PEDOT electrodes with/without lignin in LIB and NIB at different C rates. Reproduced with permission from [106,107], Copyright (2017) John Wiley and Sons, (2018) Royal Society of Chemistry.

The cathode is one of the two key redox-active materials at the positive terminal during discharge in a rechargeable battery. The quinone structure in lignin is redox-active; hence, lignin can be directly used as a cathode material in rechargeable batteries. Quinones are mobile electron carriers, and their six-membered ring with C=O functional group enables a facile and reversible two-electron redox reaction [100,101,102]. The electronic conductivity of lignin is low; hence, a conductive additive is necessary to facilitate electron transport [103]. Conductive polymers are both redox-active and conductive (electrons); therefore, combining these materials with lignin would be a strategic approach to enhance the electrochemical performance [103,104,105,106]. Lignin and PEDOT hybrid cathode was prepared by using black liquor from natural lignin and EDOT monomer for NIB and LIB (Figure 9a) [107]. The effect of the mass ratio between lignin and EDOT on electrochemical behaviors was studied. The discharge capacities tended to decrease as the mass ratio of lignin increased, and the lignin/PEDOT electrode with a 20/80 mass ratio showed better performance than the sole PEDOT electrode. The granular-cauliflower morphology of lignin/PEDOT (20/80 by wt%) confirmed by SEM has a large surface area, enabling simultaneous redox reactions at the large sites; hence, enhanced capacities are observed in both LIB and NIB (Figure 9b) [106]. The Lignin/PEDOT (20/80 by weight) in LIB demonstrates the highest capacity (92 mAh g^−1^) and retention (70%) due to improved electron transfer and possibly ion conduction of swelled PEDOT (Figure 9c). In contrast, PEDOT without lignin demonstrated 54% and 12% of reduction in specific capacity and capacity retention, respectively. Even in NIB, a similar behavior was observed. The use of lignin-coated PEDOT in LIB displayed the highest specific capacity in the first cycle, but a better rate capability was confirmed in NIB.

**Figure 10:**
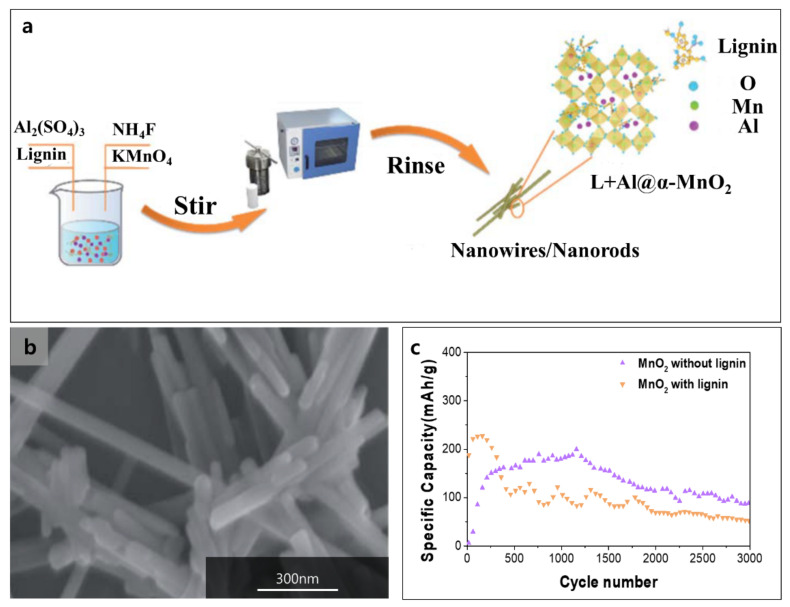
(**a**) Preparation of MnO_2_ with lignin (L+Al@a-MnO_2_) for ZIB, (**b**) SEM micrograph of MnO_2_ with lignin, and (**c**) capacity retention of MnO_2_ with/without lignin Reproduced with permission from [110], Copyright (2021) Royal Society of Chemistry.

Lignin was also used in a Zn-ion battery (ZIB) [108,109,110]. ZIB has attracted attention because of its non-toxicity, high safety, high theoretical capacity of zinc (820 mAh g^−1^), and outstanding rate capability [111,112]. However, ZIB has the limitation of the dissolution of transition metal from the cathode (i.e., Mn^2+^) and forming of zinc hydroxide sulfate (ZHS), which causes a loss of capacity [113]. To suppress them, Lignin-coated a-MnO_2_ nanorods and nanowires with Al^3+^ dopant were synthesized with NH_4_F, Al_2_(SO_4_)_3_, KMnO_4_, and sodium lignosulfonate using a hydrothermal method (Figure 10a) [110]. The MnO_2_ with lignin shows 1D nanorods providing large reaction sites (Figure 10b). Lignin helped to increase the structural stability and the area of reaction sites and Al^3+^ facilitated Zn ion transport. Through the structural stability and strong metal ion interaction of lignin, Al^3+^ is effectively doped into MnO_2_ lattice during the hydrothermal method. The doped Al^3+^ helped the access of H^+^ to control the residue of ZHS. The lignin coating on the a-MnO_2_ leads to superior electrochemical performance with high reversible capacity (180 mAh g^−1^ at 1.5 A g^−1^) and better cyclability than a-MnO_2_ that had no lignin (66.7% higher in capacity after 3000 cycles) (Figure 10c).

**Figure 11:**
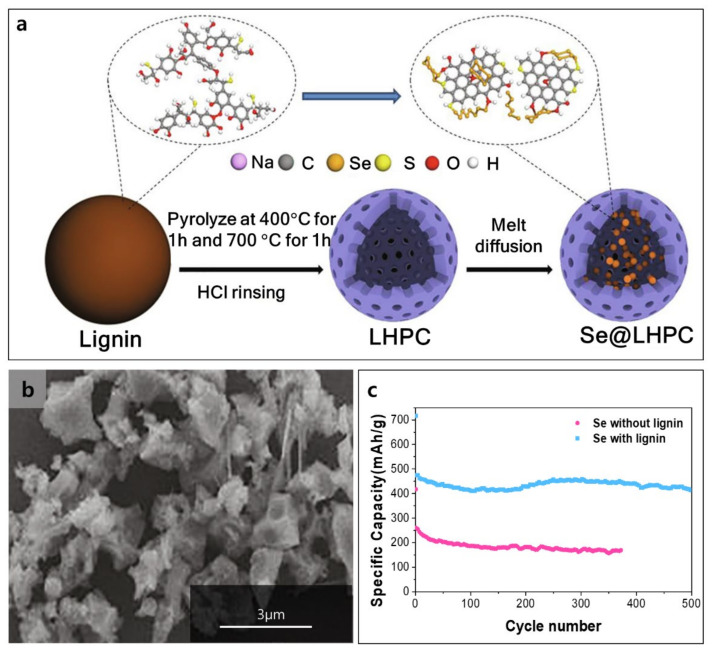
(**a**) Fabrication of Se with lignin (Se@LHPC) for Li-Se batteries, (**b**) SEM image of Se with lignin, and (**c**) cycling performance of Se with/without lignin at 0.5 C Reproduced with permission from [116], Copyright (2021) Elsevier.

Lignin can also form composite materials with other redox-active materials, enabling the production of advanced cathode materials for lithium chalcogen batteries. Chalcogen such as S, Se, and Te has low electrical conductivity; hence, the facile electron access as well as ion transport is important. Carbonized lignin is a sustainable conductor for efficient electron transport, and the heterogeneous morphology of lignin facilitates the diffusion of metal ions [114]. Furthermore, lignin has many oxygen-containing functional groups in the backbones for the adsorption of ions and redox reactions. The Li-Chalcogen battery outperforms the LIB in terms of specific and volumetric capacities [115]. The theoretical specific capacities of S, Se, and Te are 1675, 678, and 420 mAh g^−1^, and the volumetric capacities of the same are 3467, 3265, and 2621 mAh cm^−3^, respectively [116]. However, the low electronic conductivity of chalcogen and polychalcogenides dissolution/shuttle cause low active material utilization and capacity retention [117,118,119,120]. Lignin can host chalcogen materials to facilitate electron transfer and suppress polychalcogenides dissolution/shuttle [121,122,123]. Carbonized lignin and multi-walled carbon nanotube (MWCNT) composite layers could suppress polysulfides diffusion, improving S utilization and capacity retention [123]. Additionally, lignin formed a binder-free S electrode and demonstrated high capacities in the range of 1000–1400 mAh g^−1^ [124,125,126,127,128,129]. The kraft lignin was pyrolyzed to make lignin-derived hierarchical porous carbon (LHPC) and Se was introduced by melt infiltration (Figure 11a) [116]. LHPC can load large amount of Se because of hetero-atom doping, high micropore volume, and large specific surface area. Similarly, carbonized lignin combined with Te could facilitate electron transfer and ion diffusion, and endure volume change [122]. The LHPC shows a three-dimensional (3D) porous structure (Figure 11b) and Se-infiltrated LHPC shows distributed Se into the S- and O- doped porous carbon. The heteroatom-doped regions would improve the chemical adsorption of polyselenides. Lignin-based Se cathode enables to have the stable capacity of ~460 mAh g^−1^ at 0.5 C over 500 cycles and high capacity retention of 92% (Figure 11c). In comparison, the Se cathode without lignin retained only 60% of initial capacity (~180 mAh g^−1^) after 300 cycles.

**Figure 12:**
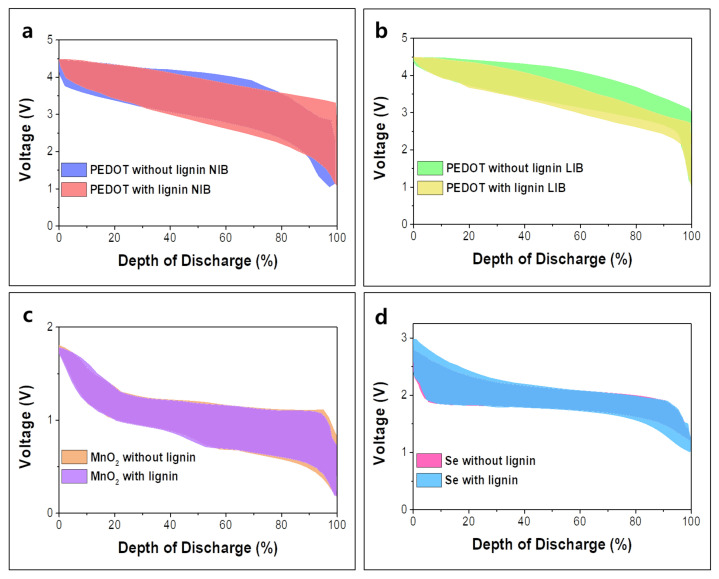
Charge–discharge profiles of PEDOT with/without lignin at C/20 in (**a**) NIB, in (b) LIB, (**c**) MnO_2_ with/without lignin at 0.1 A g^−1^ in ZIB and (**d**) Se with/without lignin at 0.5 C for Li/Se battery. Reproduced with permission from [103,107,110,116], Copyright (2018) Royal Society of Chemistry, (2021) Royal Society of Chemistry, (2021) Elsevier.

To investigate the effect of lignin on the polarization, we compared the charge–discharge profiles, as shown in Figure 12a–d [107,110,116]. Overall, the addition of lignin did not significantly influence the polarization. The lignin-coated PEDOT in LIB shows a noticeably smaller voltage difference between charge and discharge compared with the PEDOT without lignin, indicating that lignin helped to achieve higher energy efficiency. However, the output voltage of lignin-coated PEDOT is slightly lower than the sample without lignin when the state of charge becomes higher than 50%. The polarization change influenced by the lignin has not yet been studied or discussed; thus, it will be an interesting future research topic.

## 5. Challenges and Outlook

Lignin has some issues to address in the future for employment in the field of rechargeable batteries. First of all, it is confirmed that lignin itself is electrochemically active near ~0.6 V(vs Ag/AgCl). Although lignin is a promising cathode material, it cannot quickly transport electrons as it is classified as an insulator. The electronic conductivity shall be improved intrinsically or extrinsically for facile electrochemical reactions. Systematic investigations on the effect of chemical functionalization, a conductive additive, and elemental doping will be helpful to pave the way to design advanced lignin-based cathode materials. The pyrolysis of lignin produces carbon materials used in both the anode and cathode. Particularly in the anode, although lignin-based hard carbons provide high power and specific capacities, the capacity retention and Coulombic efficiency (CE) are not satisfactory. Fundamental solutions based on the understandings of capacity fade and low CE are needed. The porous carbons produced from lignin could also be used in both the cathode and anode. The pore properties (pore size distribution, pore volume, surface area, etc.) play critical roles in battery electrodes. However, up to now, lignin-derived carbons are mainly restricted to microporous carbon. Studies on the fine-tuning of pore properties of lignin-based porous carbon would render multifunctional electrodes with high ionic conductivity, selective material transport, large reaction site, foreign material storage capability, etc. Lignin and lignin-derived carbon have low densities; hence, the volumetric energy of the lignin-based anode and cathode are low. The density is an intrinsic property of material, but we can possibly modulate the density of carbonized or activated lignin by controlling the structure and pore properties of carbons. The oxygen functional groups in lignin positively influence the electrochemical performance since it enhances the redox reactions and electrolyte wetting. However, these oxygen functional groups are greatly diminished when lignin is carbonized under high temperatures and inert atmospheres. Fundamental studies relating oxygen groups and carbonization would enable the formation of high-performance anode and cathode materials. For the usage in the binder, separator, gel electrolyte, lignin shows great mechanical strength, binding ability, and ion conductivity. Lignin must be purified to remove dissoluble fractions. Dissoluble fractions may derive increased resistance and side reactions between ions. In addition, purification would increase the cost and complexity. Without solving these issues, lignin-based materials cannot be applied in portable electronics that require high gravimetric and/or volumetric energy densities. However, these problems are possibly suppressed via breakthrough research in the long term. It is important to know that no energy storage technology is satisfactory in all dimensions. The lignin-based battery materials could fit grid-scale energy storage where cost efficiency and sustainability are more important than energy density. Although only limited information is available, lignin-based materials can be also adopted in topics of inflammable electrolytes in both solid and liquid, operations in extreme conditions (low or high temperatures), recycling, cost efficiency, and non-lithium ion batteries in the future. The unique chemistry, mechanical properties, and price of lignin possibly resolve the problems in the latest research trends.

## 6. Conclusions

Lignin has distinct advantages for the components including the binder, separator, electrolyte, anode, and cathode in rechargeable batteries. The durable structure and abundant functional groups of lignin lead to great mechanical strength, binding ability, and ion conductivity, which derives improved ability in the binder, separator, and gel electrolyte. The quinone moiety in lignin is redox-active and is thus directly used as a cathode material. The functional group in lignin can suppress diffusion of conversion-type active materials, enhancing the electrochemical performances. The high fraction of carbon element in the chemical structure makes lignin an ideal carbon precursor to being used in both the anode and cathode. Lignin endures the high volume change during electrochemical reactions in the binder and prevents damage from dendrites in the separator because of its great mechanical strength. In addition, carbonization of lignin leads to a large interlayer distance for facile metal ion intercalation enabling enhanced capacity and rate capability. The chemistry of lignin can be easily designed via elemental doping and/or adsorption of the metal ions. Through these characteristics of lignin, substantial advances have been achieved in lignin-based materials for sustainable rechargeable batteries. As new strategies and applications are developed, more environmentally friendly and cheaper lignin-based materials will positively impact our lives, creating a new paradigm.
